# Differential dose-response relationships of collagen cross-linking in the cornea and sclera

**DOI:** 10.3389/fbioe.2026.1799822

**Published:** 2026-05-08

**Authors:** Haiyan Wang, Yanan Li, Lingjuan Sun, Mingqi Liu, Xiongwei Zhang, Ruibo Yang

**Affiliations:** 1 Tianjin Key Laboratory of Retinal Functions and Diseases, Tianjin Branch of National Clinical Research Center for Ocular Disease, Eye Institute and School of Optometry, Tianjin Medical University Eye Hospital, Tianjin, China; 2 Department of Ophthalmology, Shijiazhuang People’s Hospital, Shijiazhuang, Hebei, China; 3 Department of Nuclear Medicine, Shijiazhuang People’s Hospital, Shijiazhuang, Hebei, China

**Keywords:** collagen cross-linking, cornea, sclera, secant modulus, tangent modulus, Yeoh model fitting

## Abstract

**Objective:**

This study aimed to systematically compare the enhancing effects of different UVA irradiation intensities on the biomechanical properties of *ex vivo* porcine cornea and sclera, to clarify the differences in the “dose-response” relationships between the two tissues during cross-linking, and to provide a parametric basis for extending cross-linking technology to the treatment of scleral diseases such as pathological myopia.

**Methods:**

Eighty fresh *ex vivo* porcine eyes were used. The cornea and sclera were each randomly divided into 4 groups according to irradiation intensity: a control group (no irradiation), and experimental groups treated at 3,15and 30 mW/cm^2^ (total energy 5.4 J/cm^2^). Following infiltration with a 0.1% riboflavin solution, irradiation was performed with 370 nm UVA light, the procedure was assisted by pure oxygen blowing. Key parameters extracted included the secant modulus (at 2%, 6%, and 10% strain points), the tangent modulus (slope of the linear elastic region), and the Yeoh hyperelastic model parameters (C_1_, C_2_).

**Results:**

In the cornea, both the secant modulus and tangent modulus showed a increase with rising irradiation intensity, with the 30 mW/cm^2^ group exhibiting the greatest enhancement magnitude (approximately a 146.2% increase in secant modulus at 10% strain). For the sclera, the modulus enhancement displayed a non-linear relationship, the 15 mW/cm^2^ group showed the optimal reinforcement effect (approximately a 176.13% increase in secant modulus), while the effect diminished in the 30 mW/cm^2^ group. Analysis of model parameters indicated that the increase in C_2_ (governing nonlinear stiffening) after cross-linking was far greater than that in C_1_ (governing initial stiffness), suggesting that cross-linking primarily enhances the tissue’s resistance under large deformations. Inter-group comparisons revealed that under identical parameters at low to medium intensities (3 and 15 mW/cm^2^), the sclera exhibited a higher modulus increase rate than the cornea; at high intensity (30 mW/cm^2^), the cornea showed more significant enhancement.

**Conclusion:**

The responses of the cornea and sclera to cross-linking intensity are fundamentally different: the reinforcement effect in the cornea increases with intensity, whereas the sclera has an optimal intensity (15 mW/cm^2^), beyond which further intensity increase leads to diminishing returns.

## 1 Introduction

The cornea and sclera constitute the core load-bearing structure of the eyeball wall, whose biomechanical integrity is crucial for maintaining ocular morphology and enabling normal visual function. Both corneal diseases (such as keratoconus) and scleral diseases (such as progressive myopia and scleromalacia) are closely associated with the structural weakening of collagen fibers, with the core pathophysiological change being a progressive decline in biomechanical strength. Riboflavin/ultraviolet A (UVA)-induced collagen cross-linking treatment is an established clinical intervention that reinforces the corneal stroma and arrests the progression of keratoconus (Wollensak et al., 2003; Raiskup and Spoerl, 2011). The underlying mechanism is initiated by the photoexcitation of riboflavin upon exposure to UVA light (peak absorption 370 nm). This excited state primarily drives a Type II photochemical reaction, generating reactive oxygen species (ROS), notably singlet oxygen (McCall et al., 2010; Kamaev et al., 2012). These highly reactive molecules then catalyze the formation of novel covalent bonds between adjacent collagen molecules and microfibrils within the stromal extracellular matrix via oxidative post-translational modifications. This process creates supplementary cross-links that fortify the extant collagen network. On a microstructural level, the augmented cross-linking density curtails interfibrillar sliding and the rearrangement of collagen lamellae under biomechanical stress, thereby substantially enhancing the tissue’s rigidity and its resistance to enzymatic degradation (Hayes et al., 2013; Spoerl et al., 2004; Dias et al., 2013).

Existing standard cross-linking protocols (Dresden protocol: 3 mW/cm^2^ for 30 min, total energy 5.4 J/cm^2^) were primarily optimized and established based on corneal light transmissibility, thickness, and endothelial cell tolerance, which leads to patient discomfort, prolonged operating time, and risks of corneal dehydration. These limitations have driven the clinical demand for accelerated CXL protocols that deliver the same total energy in a shorter time by increasing UVA irradiance. Such protocols could significantly improve patient comfort, streamline clinical workflows, and reduce intraoperative complications (Wen et al., 2018; Mazzotta et al., 2021). The sclera exhibits significant differences from the cornea in terms of structural composition (collagen types, arrangement), thickness, pigment content, and light transmissibility. These differences directly affect the penetration depth and energy distribution of light within the tissue. Consequently, directly applying corneal cross-linking parameters to the sclera may be inefficient or uncertain. Currently, there is a lack of systematic comparative studies on the differential effects of cross-linking treatments with different energy intensities on the biomechanical enhancement of the cornea versus the sclera. Clarifying this “dose-effect” relationship is crucial for expanding the clinical applications of cross-linking technology (treating pathological myopia, scleral reinforcement) and developing personalized treatment strategies.

Therefore, this study aims to systematically investigate the enhancing effects of different UVA irradiation intensities (with constant total energy) on the biomechanical properties of corneal and scleral samples. By comparatively analyzing the mechanical responses of the two tissue types under different cross-linking “doses”, we seek to identify their respective optimal reinforcement parameters. This will provide direct experimental evidence for future refined and differentiated cross-linking treatments targeting various ocular diseases.

## 2 Materials and methods

### 2.1 Experimental design and grouping

Experimental Animals and Sample Preparation: A total of 80 fresh *ex vivo* porcine eyeballs, obtained within 6 h post-mortem, were used in this study. The animal study was approved by Experimental Animal Welfare and Ethics Committee of Shijiazhuang People’s Hospital [2025(007)]. Porcine eyes are widely used in ocular biomechanical research due to their high similarity to human eyes in size, structure, and collagen composition. The study employed an *ex vivo* controlled design. Based on different UVA irradiation intensities, corneal and scleral samples were randomly assigned to 8 experimental groups respectively. *A priori* power analysis (effect size d = 0.8, α = 0.0125, power = 0.80) indicated a required sample size of approximately 9 per group; therefore, 10 samples per group were used to ensure adequate statistical power and account for potential technical failures (Spoerl et al., 1998; Wollensak et al., 2014). (see Table 1).

**TABLE 1:** Group of cornea and sclera by different irradiation intensities.

Group	Number	Irradiation intensity
GroupA (CCG)	10	No irradiation
GroupB(CXL1)	10	3 mW/cm^2^ 30 min
GroupC(CXL2)	10	15 mW/cm^2^6 min
GroupD (CXL3)	10	30 mW/cm^2^3 min
GroupE (SCG)	10	No irradiation
GroupF(SXL1)	10	3 mW/cm^2^ 30 min
GroupG (SXL2)	10	15 mW/cm^2^6 min
GroupH(SXL3)	10	30 mW/cm^2^3 min

Groups A to D are the corneal cross-linking groups (CXL), while Groups E to H are the scleral cross-linking groups (SXL).

### 2.2 Experimental procedure

Corneal Cross-linking Procedure: The eyeballs were thoroughly rinsed with saline solution to remove any debris, and the extraocular muscles were dissected away. The central corneal epithelium was gently removed using a sterile alcohol swab to expose Bowman’s layer. Immediately after epithelial removal, riboflavin soaking was initiated: 0.1% riboflavin solution (PESCHKE-M Rapid, containing 0.1% Riboflavin (Vitamin B2) and 1.0% HPMC) was applied for corneal saturation. One drop of the riboflavin solution was instilled every 2 min for a total duration of 30 min. Subsequently, UVA irradiation was performed using a UVA irradiation device (Model: i-MYO XL01, Super Vision Technology, Inc., Beijing, China). This integrated device (i-MYO XL01) uniquely combines three delivery channels—UVA irradiation, riboflavin perfusion, and oxygen supply into a single probe system. The irradiation parameters were set and adjustable within the experiment, with an irradiance range of 3–30 mW/cm^2^. During irradiation, one drop of riboflavin solution was supplemented onto the corneal surface every 5 min to maintain a stable saturation level. Concurrently, 100% pure oxygen was continuously blown onto the ocular surface (flow rate approximately one bubble per second) to enhance oxygen participation in the photochemical reaction. Upon completion of irradiation, all samples were thoroughly rinsed with PBS. Central corneal thickness was measured using an ultrasonic pachymeter (Tomey SP-100, Japan). A complete circular incision was made along the corneal limbus using a surgical blade, and the entire cornea was carefully separated and removed. It was then placed on a corneal cutting block, and a standardized corneal strip (3 mm wide, 23 mm long) was prepared using a dedicated corneal cutting tool.

Scleral Cross-linking Procedure: The eyeballs were thoroughly rinsed with saline solution, and the extraocular muscles were dissected away. The superior-temporal quadrant of the sclera at the equatorial region (between the superior rectus and lateral rectus muscles) was exposed. The riboflavin soaking and irradiation procedures were identical to those described for the cornea. After irradiation, all samples were thoroughly rinsed with PBS. To preserve the fiber orientation, the sclera was cut along the direction of the ocular axis (from the cornea towards the optic nerve) into strips measuring 3 mm in width and 23 mm in length. Scleral thickness was measured using a thickness gauge.

Core Variable Control: To investigate the intensity effect, the total irradiation energy was uniformly set at 5.4 J/cm^2^. The irradiation time for each group was adjusted using the formula “Irradiation time (seconds) = Total energy (J/cm^2^)/Intensity (W/cm^2^)” to ensure a consistent total energy delivery. Irradiation was performed using a contact probe with a spot size of 8 mm in diameter, ensuring direct contact between the light source and the sample surface.

**FIGURE 1:**
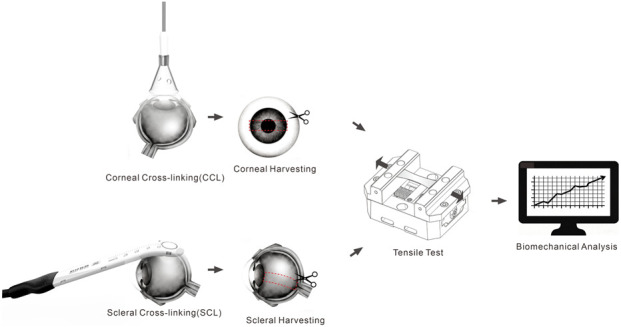
Flowchart of the porcine eyeball cross-linking procedure.

After preparation, the corneal and scleral strips were ready for biomechanical testing. All tensile tests were performed with specimens fully immersed in a temperature-controlled physiological saline bath maintained at 37 °C. The test was conducted using a computer-controlled biomaterial testing machine (Tianjin Kail, IBTC-300). The gauge length between the clamps was set at 6 mm. A preload of 0.02 N was first applied, followed by uniaxial tensile testing at a constant speed of 0.025 mm/s. The test was stopped when the load reached 10 N or the tensile displacement reached 5 mm, and the stress-strain data were recorded. Engineering stress and strain were used for all mechanical analyses. Stress was calculated as σ = F/A_0_, where F is the measured force and A_0_ is the initial cross-sectional area (width × thickness). Strain was calculated as ε = ΔL/L_0_, where ΔL is the displacement and L_0_ is the initial gauge length (6 mm). Corneal thickness was measured *in situ* using an ultrasonic pachymeter prior to excision, and strip width was measured using a digital caliper prior to testing. A detailed flowchart is presented in Figure 1.

### 2.3 Data processing

#### 2.3.1 Elastic modulus

To comprehensively quantify the stiffness characteristics of the tissues, this study extracted and calculated two key elastic modulus parameters.

1. Secant Modulus: Defined as the slope of the line connecting the origin (0% strain) to a specific point on the stress-strain curve (at 2%, 6%, and 10% strain). It reflects the overall, average stiffness of the tissue within the deformation range from its initial state to that specific strain level. This serves as a macroscopic indicator for evaluating the material’s overall resistance to deformation during that specific stage of deformation.
2. Tangent Modulus: Defined as the instantaneous slope (the first derivative) of the stress-strain curve at a specific strain point. It characterizes the rate of change in stiffness of the tissue at that instantaneous deformation state. This parameter is particularly sensitive for revealing the hardening or softening behavior of the material in the nonlinear deformation region (at larger strains) and can more precisely reflect the immediate response of the material’s microstructure.

#### 2.3.2 Material parameter fitting based on a hyperelastic constitutive model

To deeply and quantitatively characterize the impact of cross-linking on the micromechanical properties of the cornea and sclera, going beyond traditional single-modulus analysis, this study further employed a hyperelastic constitutive model to fit the obtained stress-strain data. This method uses a set of material constants with physical significance to comprehensively describe the nonlinear, incompressible mechanical behavior of the tissue across the entire large-deformation range, thereby more precisely quantifying the differential effects of cross-linking treatment on tissue stiffness at different deformation stages.

Model Selection and Formulation: The Yeoh model (Yeoh, 1993), widely used for describing biological soft tissues, was selected for fitting in this study. The strain energy density function W for this model is expressed as:

$$W=\sum_{i=1}^{n}C_{i}{\left(I_{1}-3\right)}^{i}+\sum_{k=1}^{i}\frac{1}{D_{k}}{\left(J-1\right)}^{2k}$$

where I_1_ is the first invariant of the Cauchy-Green strain tensor, and C_1_, C_2_ are the material constants to be fitted. This model is suitable for describing the uniaxial tensile behavior of incompressible materials (J = 1). Under uniaxial tensile conditions, the principal stretch ratios are λ_1_ = λ (in the tensile direction) and λ2 = λ3 = λ^−1/2^ (in the transverse contraction directions). The corresponding engineering stress can be derived as the derivative of W with respect to λ.

Data Preparation and Fitting Process: The fitting analysis was based on the original uniaxial tensile data. For each experimentally obtained engineering stress vs. stretch ratio (λ = 1 + ε) curve, nonlinear least squares fitting was performed using MATLAB (version R2023a, MathWorks). The optimization objective was to minimize the sum of squared residuals between the model-predicted stress and the experimental stress. To ensure fitting stability and physical plausibility, the range of the stretch ratio \lambda was limited to correspond to a strain range of 0%–10%.

Through the above process, a unique set of material constants (C_1_, C_2_) was obtained for each experimental sample. C_1_primarily governs the mechanical response at small strains (the linear region), while C_2_ governs the response at large strains (the nonlinear hardening region).

#### 2.3.3 Statistical methods

The stress-strain data obtained from the experiments were automatically recorded by the testing system software. For each curve, key mechanical parameters, namely, Elastic Modulus, were calculated: the secant modulus at specific strain points (2%, 6%, and 10%), and the tangent modulus within the linear elastic phase (strain range of 1%–10%). All data are presented as mean ± standard deviation.

Statistical analysis was performed using software such as GraphPad Prism. The data were first tested for normality (Shapiro-Wilk test) and homogeneity of variance (Levene’s test). When the assumptions for parametric tests were met, inter-group comparisons were conducted using one-way analysis of variance (One-way ANOVA). To compare the performance differences between the cornea and sclera under identical parameters, paired-samples t-tests were used if both normality and homogeneity of variance were satisfied; otherwise, the Wilcoxon signed-rank test was employed. To account for multiple comparisons across treatment groups, Bonferroni correction was applied. For intra-tissue comparisons among four groups (cornea: A, B, C, D; sclera: E, F, G, H), a total of six independent tests were conducted. The significance level was adjusted to α = 0.05/6 = 0.0083 for these comparisons. For inter-tissue comparisons of percentage changes (B vs. F, C vs. G, D vs. H), three independent tests were performed, with a corrected significance level of α = 0.05/3 = 0.0167. Adjusted p-values are reported where applicable.

## 3 Results

### 3.1 Sample thickness measurements

Prior to biomechanical testing, corneal and scleral thickness were measured. The mean corneal thickness across all groups ranged from 1.176 to 1.202 mm, and the mean scleral thickness ranged from 0.929 to 1.235 mm. No statistically significant differences in baseline thickness were observed among corneal groups and scleral groups (one-way ANOVA, all adjusted P > 0.05). See Table 2.

**TABLE 2:** Sample thickness measurements.

Group	Thickness (mm)	p
GroupA	1.184 ± 0.037	0.379
GroupB	1.202 ± 0.034	
GroupC	1.189 ± 0.035	
GroupD	1.176 ± 0.026	
Group E	1.115 ± 0.215	0.097
Group F	0.929 ± 0.240	
Group G	1.195 ± 0.321	
Group H	1.235 ± 0.293	

### 3.2 Corneal biomechanical changes

**FIGURE 2:**
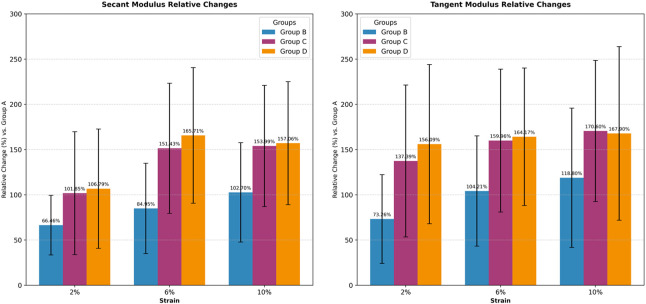
Relative changes in corneal biomechanical properties (vs. Group A).

Corneal cross-linking significantly enhanced biomechanical properties across all treatment groups compared to the control (group A). At 2%, 6%, and 10% strain, both the secant modulus and tangent modulus of groups B (3 mW/cm^2^), C (15 mW/cm^2^), and D (30 mW/cm^2^) were significantly higher than those of group A (all adjusted P < 0.083). However, no statistically significant differences were detected among the three treatment groups for either modulus at any strain level (all adjusted P > 0.0083). See Table 3 and Figure 2.

**TABLE 3:** Biomechanical changes in corneal cross-linking.

​	​	2%	6%	10%
Secant modulus	Group A	0.486 ± 0.140	0.525 ± 0.182	0.815 ± 0.315
	Group B	0.809 ± 0.196*	0.971 ± 0.335*	1.652 ± 0.543*
	Group C	0.981 ± 0.498*	1.320 ± 0.512*	2.070 ± 0.712*
	Group D	1.005 ± 0.474*	1.395 ± 0.513*	2.095 ± 0.657*
Tangent modulus	Group A	0.460 ± 0.164	0.974 ± 0.420	2.000 ± 1.054
	Group B	0.797 ± 0.298*	1.989 ± 0.728*	4.376 ± 1.807*
	Group C	1.092 ± 0.550*	2.532 ± 0.931*	5.412 ± 2.136*
	Group D	1.178 ± 0.577*	2.573 ± 0.871*	5.358 ± 2.201*

*P < 0.0083 compared between each treatment group (B, C, D) and control group A for both secant modulus and tangent modulus at 2%, 6%, and 10% strain.

### 3.3 Scleral biomechanical changes

**FIGURE 3:**
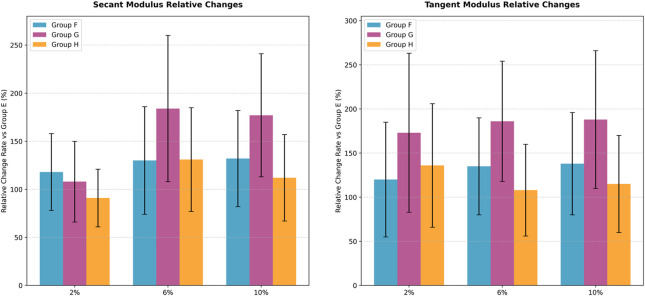
Relative changes in scleral biomechanical properties (vs. Group E).

Similarly, scleral cross-linking significantly increased both moduli in all treated groups (F, G, H) relative to the control (group E) at 2%, 6%, and 10% strain (all adjusted P < 0.083). Pairwise comparisons among groups F, G, and H revealed no significant differences in either secant or tangent modulus at any strain level (all adjusted P > 0.0083). See Table 4 and Figure 3.

**TABLE 4:** Biomechanical changes in scleral cross-linking.

​	​	2%	6%	10%
Secant modulus	GroupE	1.153 ± 0.346	2.509 ± 1.146	4.966 ± 2.019
	GroupF	2.512 ± 0.918*	5.749 ± 2.296*	11.474 ± 3.752*
	GroupG	2.392 ± 1.074*	7.112 ± 2.437*	13.712 ± 4.113*
	GroupH	2.194 ± 0.735*	5.769 ± 2.095*	10.554 ± 3.774*
Tangent modulus	Group E	1.604 ± 0.861	6.191 ± 2.775	15.441 ± 7.217
	Group F	3.531 ± 1.834*	14.521 ± 5.200*	36.700 ± 12.864*
	Group G	4.373 ± 2.089*	17.657 ± 4.201*	44.456 ± 14.004*
	Group H	3.7838 ± 1.874*	12.910 ± 5.846*	33.182 ± 15.144*

*P < 0.0083 compared between each treatment group (F, G, H) and control group E for both secant modulus and tangent modulus at 2%, 6%, and 10% strain.

### 3.4 Inter-tissue comparison of cross-linking efficacy

To compare the relative efficacy of cross-linking between the cornea and sclera under identical irradiation parameters, the percent change in modulus relative to respective controls was analyzed. For the 3 mW/cm^2^ (B vs. F) and 15 mW/cm^2^ (C vs. G) comparisons, the data met normality and homogeneity of variance assumptions, allowing paired t-tests. For the 30 mW/cm^2^ comparison (D vs. H), where homogeneity of variance was violated at certain strains, the Wilcoxon signed-rank test was used. In the secant modulus, the comparison between group F and group B, as the strain level increases, the rate of change of the secant modulus becomes higher, with group F exhibiting a greater change rate than group B. Similarly, in the comparison between group C and group G, group G shows a higher rate of change in the secant modulus than group C. However, the trend is reversed for groups D and H, where group H demonstrates a lower rate of change compared to group D. The heatmap illustrates that the differences in secant modulus change rates between the cornea and sclera were not statistically significant for all group comparisons (adjusted P > 0.0167).

**FIGURE 4:**
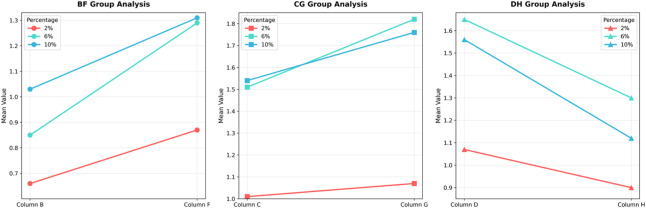
Comparison of Biomechanical Change Rates in secant modulus. The line chart illustrates the trends in the rate of change of the secant modulus for the cornea and sclera at different strain levels.

**FIGURE 5:**
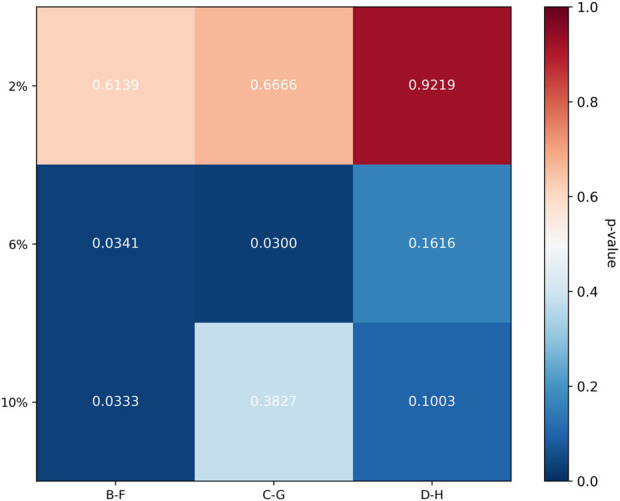
Heatmap of p-Values for Differences in Secant Modulus Change Rates. The heatmap illustrates that the differences in secant modulus change rates between the cornea and sclera were not statistically significant for all group comparisons (adjusted P > 0.0167).

**FIGURE 6:**
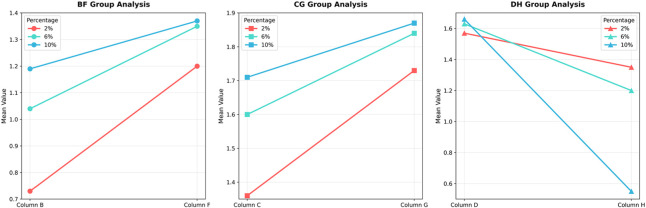
Comparison of Biomechanical Change Rates in the tangent modulus, the line chart illustrates the trends in the rate of change of the tangent modulus for the cornea and sclera at different strain levels.

**FIGURE 7:**
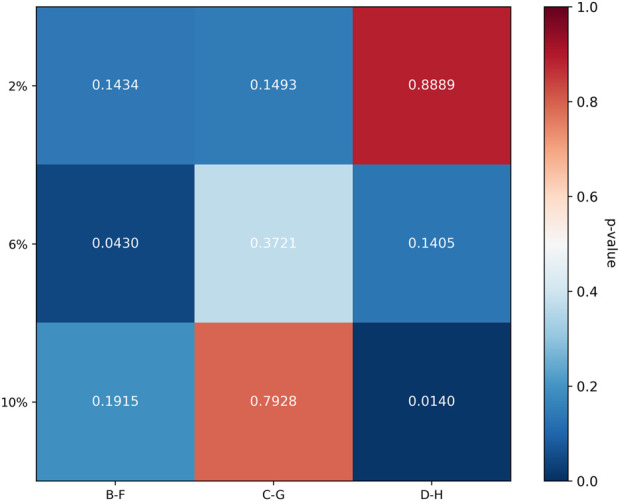
Heatmap of p-Values for Differences in Tangent Modulus Change Rates the difference in tangent modulus change rates between groups D and H at 10% strain was statistically significant (adjusted P < 0.0167). No other comparisons reached statistical significance.

For the tangent modulus, The trend is generally consistent with the pattern observed for the secant modulus, the difference in tangent modulus change rates between groups D and H at 10% strain was statistically significant (adjusted P < 0.0167). No other comparisons reached statistical significance. The complete statistical results for both secant and tangent modulus are summarized in Figures 4–7.

### 3.5 Fitting results

#### 3.5.1 Intragroup fitting results for the cornea

Comparisons between experimental groups B, C, D and control group A revealed that the experimental intervention significantly altered the mechanical response of the tissue. For the C_2_ parameter, all three treatment groups showed significantly higher values compared to group A after Bonferroni correction (adjusted P > 0.0083), with P-values of 0.00483 (B vs. A), 0.001621 (C vs. A), and 0.005256 (D vs. A). For the C_1_ parameter, no significant differences were observed between any treatment group and control after correction. Additionally, no statistically significant differences were detected among the three treatment groups for either C_1_ or C_2_ (adjusted P > 0.0083).

#### 3.5.2 Intragroup fitting results for the sclera

Comparison between experimental groups F, G, H and control group E revealed that the experimental intervention significantly altered the mechanical response of the tissue, C_2_ values in groups G and H were significantly higher than those in group E, with P-values of 0.00005 (G vs. E) and 0.00215 (H vs. E). For the C_1_ parameter, no significant differences were observed between any treatment group and control after correction. Additionally, no statistically significant differences were detected among the three treatment groups (F vs. G, F vs. H, G vs. H) for either C_1_ or C_2_ (adjusted P > 0.0083).

#### 3.5.3 Intergroup fitting results between cornea and sclera

The p-values for the normality tests of the C-value differences between groups A and E, B and F, C and G, as well as D and H were all greater than 0.05. Therefore, the differences in both C_1_ and C_2_ values for the pairs A/E, B/F, C/G, and D/H followed a normal distribution, meeting the prerequisite for conducting paired-samples t-tests.

In the difference tests, the p-values for both the C_1_ and C_2_ values in the pairs A/E, B/F, C/G, and D/H were all less than 0.05. Consequently, the differences in C_1_ and C_2_ values between the cornea and sclera were statistically significant. See Table 5.

**TABLE 5:** Analysis results of differences in fitted C-values between cornea and sclera (p-values).

​	​	A-E	B-F	C-G	D-H
Normality test	C_1_	0.1911	0.8881	0.5076	0.0604
	C_2_	0.3153	0.1292	0.4526	0.8243
t-test	C_1_	0.0259	0.0191	0.0154	0.0147
	C_2_	0.0004	0.0000	0.0000	0.0001

### 3.6 Average stress-strain curves and Yeoh model fitting

**FIGURE 8:**
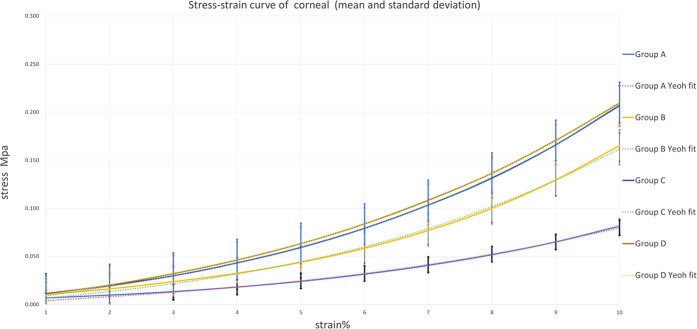
Average Corneal stress-strain curves with Yeoh model fits. Groups A, B, C and D, dashed lines represent Yeoh model fits. All CXL-treated groups (B,C and D) exhibited progressively upward-shifted curves compared to the control group (A), Yeoh model fits closely align with the experimental curves.

**FIGURE 9:**
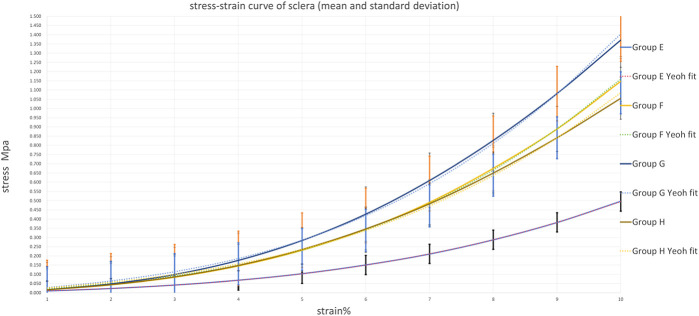
Average Scleral stress-strain curves of scleral with Yeoh model fits. Groups E, F, G and H, dashed lines represent Yeoh model fits. All CXL-treated groups (F,G and H) exhibited progressively upward-shifted curves compared to the control group (E), group G showed the most pronounced upward shift. Yeoh model fits closely align with the experimental curves.

To visually demonstrate the biomechanical changes induced by different CXL protocols and validate the constitutive modeling approach, representative stress-strain curves with Yeoh model fits are presented in Figures 8, 9. For the cornea (Figure 8), all CXL-treated groups (B, C, and D) exhibited progressively upward-shifted curves compared to the control group (A), indicating increased stiffness with higher irradiance. The excellent agreement between experimental data and model fits (*R* ^2^ > 0.98) confirms the suitability of the Yeoh model for describing corneal biomechanical behavior. For the sclera (Figure 9), group G showed the most pronounced upward shift, while group H displayed a slight reduction compared to group G. This non-linear pattern suggests an optimal intensity window for scleral cross-linking. The close alignment between experimental curves and fitted models further validates the constitutive approach across all treatment groups.

## 4 Discussion

Collagen cross-linking technology has become an established clinical approach for enhancing the biomechanical properties of the cornea and halting the progression of keratoconus. However, when extending its application to scleral reinforcement for managing conditions such as pathological myopia, it is imperative to re-evaluate the “dose-effect” relationship. This necessity arises from the fundamental differences in tissue architecture, optical properties, and biomechanical behavior between the cornea and sclera (Komai and Ushiki, 1991; Keeley et al., 1984; Lewis et al., 2016). As a transparent refractive medium, the corneal stroma is composed of highly regular lamellae of type I and V collagen fibers, ensuring high light transmittance. In contrast, the sclera serves as an opaque protective shell, consisting primarily of coarse, interwoven bundles of type I and III collagen fibers, forming a denser and more irregular structure to maintain ocular morphology and resist external forces (Coudrillier et al., 2015). For the first time under the condition of constant total energy (5.4 J/cm^2^), this study systematically compared the biomechanical enhancement effects of different UVA irradiances on the cornea and sclera. By integrating three analytical methods—the secant modulus, tangent modulus, and hyperelastic constitutive model (Yeoh model)—it reveals the differential response mechanisms of these two tissues to cross-linking stimulation from macro-to micro-levels.

In the cornea, examination of the stress-strain curves revealed a trend toward greater stiffening with increasing irradiance. However, no statistically significant differences in elastic modulus were detected among the three treatment groups. These results demonstrate that, under constant total energy (5.4 J/cm^2^) and with adequate oxygen supplementation, all accelerated protocols (15 and 30 mW/cm^2^) achieve mechanical stiffening comparable to the standard Dresden protocol (3 mW/cm^2^).

A critical consideration when comparing CXL outcomes is the distinction between irradiance (intensity, mW/cm^2^, applied for a specific irradiation time such as 30 min for standard protocols or 6 min and 3 min for accelerated protocols) and total energy fluence (J/cm^2^), as both parameters influence the final biomechanical effect. Several studies have systematically examined the effect of total energy fluence on corneal stiffening. Fischinger et al. (2023) evaluated porcine corneas treated with 18 mW/cm^2^ at varying fluence levels (5.4, 10.8, 15, and 20 J/cm^2^) and found that stress at 10% strain increased by 31%, 52%, 56%, and 76%, respectively, compared to controls. Similarly, Boschetti et al. (2021) applied 9 mW/cm^2^ with exposure times of 2.5–20 min (fluence 1.35–10.8 J/cm^2^) and reported that stiffness gain generally increased with fluence, although no significant differences were observed among groups with ≤10 min exposure (≤5.4 J/cm^2^). Liu et al. (2020) further quantified the hyper-viscoelastic properties of human corneal stroma after CXL with different energies (0–5.4 J/cm^2^), demonstrating that increasing UVA energy dose significantly enhanced corneal strength and hyperelastic stiffness (the C_2_ parameter), while reducing maximum stretch and viscosity. Collectively, these studies establish that increasing total fluence produces greater stiffening.

Regarding high-irradiance/short-duration protocols with constant total energy, the literature presents varied findings. Bao et al. (2018) systematically compared irradiances ranging from 3 to 90 mW/cm^2^ (all at 5.4 J/cm^2^) in rabbit corneas and reported that the stiffening effect decreased with increasing irradiance, with significant effects only observed at 3 and 9 mW/cm^2^. The authors concluded that the Bunsen-Roscoe law (the principle of reciprocity of intensity and time) may not be readily applicable to corneal CXL, likely due to oxygen diffusion limitations. In contrast, Abrishamchi et al. (2021) found that an accelerated high-fluence protocol (18 mW/cm^2^ for 9′15″, 10 J/cm^2^) achieved stiffening comparable to the standard Dresden protocol (3 mW/cm^2^ for 30 min, 5.4 J/cm^2^). This suggests that higher fluence can compensate for the reduced efficacy of accelerated protocols when total energy is increased, rather than held constant.

Our findings align with and extend these observations. Consistent with Coudrillier et al. (2015), we observed no statistically significant differences among irradiance groups when total energy was held constant at 5.4 J/cm^2^. However, a key distinction of our study is the continuous oxygen supplementation provided during irradiation. This methodological feature likely explains why, in contrast to the diminished efficacy reported by Bao et al. at higher irradiances, all accelerated protocols in our study achieved mechanical stiffening comparable to the standard Dresden protocol, with no reduction in efficacy at 30 mW/cm^2^.

This interpretation is supported by the literature on oxygen kinetics. Richoz et al. (2013) demonstrated that accelerated CXL protocols (7 mW/cm^2^ for 15 min) resulted in significantly less stiffening than the standard protocol, attributing this to oxygen depletion during the rapid consumption of oxygen at higher irradiances. The authors emphasized that oxygen availability is a rate-limiting factor in the photochemical reaction, and that insufficient replenishment during short irradiation times compromises cross-linking efficacy. Our results directly address this limitation. By providing continuous surface oxygenation, we ensured adequate stromal oxygen availability even at the highest irradiance of 30 mW/cm^2^. This is corroborated by recent work from Menzel-Severing et al. (2024), who reported that supplementary oxygen increases stromal oxygen concentration during CXL and eliminates the oxygen bottleneck at higher irradiances, thereby enabling potentially more efficient cross-linking. Our results demonstrate that when total energy is maintained at 5.4 J/cm^2^ and oxygen is adequately supplemented, accelerated CXL protocols achieve mechanical outcomes comparable to the standard Dresden protocol. The integrated design of our delivery system—ensuring simultaneous and precise administration of UVA light, riboflavin, and oxygen—thus represents a potentially novel approach for optimizing accelerated CXL protocols.

The sclera’s response displayed pronounced nonlinearity. Data from both the secant and tangent moduli consistently indicated that 15 mW/cm^2^ (Group G) was the optimal intensity for scleral reinforcement, yielding the greatest enhancement at 6% and 10% strain. However, when the intensity was increased to30 mW/cm^2^ (Group H), the strengthening effect decrease. This “inverted V-shaped” relationship can be attributed to the sclera’s thicker, denser structure, which is rich in pigment and features interwoven collagen fibers. Lower intensity (3 mW/cm^2^) may result in insufficient UVA penetration depth, limiting the efficacy of cross-linking. Conversely, excessively high intensity (30 mW/cm^2^) may cause superficial tissue over-cross-linking or induce thermal effects, potentially hindering energy transfer to deeper layers or affecting oxygen diffusion, thereby reducing the overall cross-linking efficiency. The literature shows that at an irradiance of 6 mW/cm^2^, mechanical strength at 8% strain can increase significantly, by up to 143% ± 92%. Furthermore, 4.2 mW/cm^2^ UVA irradiation was reported to increase the biomechanical strength of the posterior equatorial sclera in rabbit eyes by 464.7% (Gawargious et al., 2020; Wollensak et al., 2005; Villegas et al., 2024; Schuldt et al., 2015; Rong et al., 2017). The results of this study directly demonstrate that extrapolating the standard Dresden parameters, optimized for the cornea, directly to the sclera is inadequate. This underscores the necessity of identifying independent and optimized cross-linking parameters specifically for the sclera.

Hyperelastic model parameters (C_1_, C_2_) reveal the mechanism by which cross-linking enhances nonlinear stiffening capability at the constitutive level. While traditional single-point modulus analysis can reveal macroscopic trends, it is difficult to quantitatively distinguish the effects of cross-linking on different stages of tissue deformation. This study introduced Yeoh hyperelastic model fitting, obtaining material constants C_1_ and C_2_ with clear physical significance. Analysis revealed that the C_1_ values for both tissues increased significantly after cross-linking. This corresponds to the notable increase in the secant modulus at low strain (2%), together demonstrating that cross-linking effectively enhances the tissue’s initial linear stiffness. This enhancement primarily stems from the newly formed inter-collagen covalent bonds strengthening the overall connectivity of the fiber network (Depalle et al., 2015; Gouissem et al., 2022; Buehler, , 2006; Shen et al., 2008). A more insightful finding lies in the change of the C_2_ value, which governs the material’s nonlinear stiffening response at large strains. In this study, for both the cornea and sclera, the effective cross-linking groups (corneal Groups C and D, scleral Group G) exhibited a far greater increase in C_2_ values compared to C_1_. This constitutive-level result perfectly explains the core phenomenon observed in the macroscopic mechanical data: the percentage increase in both the secant and tangent moduli was much higher at high strains (6%, 10%) than at low strain points. This indicates mechanistically that the core biomechanical benefit of collagen cross-linking is not merely a simple “stiffening” but a significant enhancement of the tissue’s ability to resist further stretching during large deformations (the nonlinear stiffening response). This is likely because cross-linking restricts excessive sliding and rearrangement of collagen fibers, allowing the fiber network to bear load more effectively in the later stages of stretching.

The observed differential responses between corneal and scleral tissues under identical parameters may suggest that scleral collagen possesses higher photochemical sensitivity or superior energy utilization efficiency within specific UVA intensity ranges. This tissue-specific behavior could be attributed to differences in collagen composition, fibril architecture, or optical properties between the two tissues, and warrants further investigation. However, under the condition of 30 mW/cm^2^, the situation reversed, with the cornea (Group D) demonstrating significantly higher reinforcement efficiency than the sclera (Group H). This further supports the conclusion of an “optimal intensity window for the sclera” and indicates that excessively high irradiance may lead to diminishing returns for scleral reinforcement. From a clinical translation perspective, this study provides parameter guidance for differentiated treatment strategies: For corneal cross-linking, within safety thresholds, higher irradiance (30 mW/cm^2^) may yield superior mechanical outcomes, aligning with the clinical exploration direction of accelerated corneal cross-linking (Accelerated CXL) (Chow et al., 2015; Tomita et al., 2014; Ulusoy et al., 2017). For scleral reinforcement, 15 mW/cm^2^ (total energy 5.4 J/cm^2^) is the recommended parameter within this experimental framework, providing crucial evidence for future preclinical research on scleral cross-linking.

This study has several limitations. First, it employed an *ex vivo* porcine eye model which, despite similarities to human eyes, lacks *in vivo* biological healing responses and the sustained influence of intraocular pressure. Second, regarding constitutive modeling, the Yeoh hyperelastic model assumes material isotropy, which simplifies the pronounced anisotropic structure of corneal and scleral collagen architecture. Third, mechanical testing involved uniaxial quasi-static loading that does not fully simulate multi-axial dynamic *in vivo* conditions. Fourth, the research focused on immediate biomechanical effects without assessing long-term histological changes or optical impacts. Fifth, corneal thickness values were higher than previously reported physiological values, suggesting some degree of swelling under the experimental conditions, which may be attributed to the use of isotonic saline during testing.

Future studies should address these limitations by: 1) validating optimal parameters using *in vivo* animal models to incorporate biological healing responses; 2) employing inverse finite element modeling with fiber-reinforced constitutive laws (e.g., Holzapfel-Gasser-Ogden model) to better capture tissue anisotropy; 3) utilizing more complex mechanical testing methods such as inflation testing and dynamic mechanical analysis; 4) integrating long-term histological and biochemical analyses to assess cross-linking depth, collagen remodeling, and optical property changes; and 5) using a hyperosmotic solution (e.g., 5%–10% Dextran) in the hydration bath to better control tissue hydration and maintain physiological thickness in future *ex vivo* studies.

## 5 Conclusion

By integrating analyses of the secant modulus, tangent modulus, and hyperelastic constitutive modeling, this study systematically elucidates that: the biomechanical reinforcement effect of the cornea shows an increasing trend with higher UVA irradiance, whereas the sclera exhibits a non-monotonic dose-effect relationship, with peak reinforcement at 15 mW/cm^2^ followed by a decline at 30 mW/cm^2^, indicating an optimal intensity window for scleral cross-linking. The core mechanism of cross-linking lies in significantly enhancing the tissue’s stiffening capacity (marked by a sharp increase in the C_2_ value). This research strongly demonstrates that cross-linking therapy for the cornea and sclera requires parameter optimization based on tissue-specific characteristics, thereby laying a solid experimental foundation for future refined collagen cross-linking treatments of ocular diseases.

## Data Availability

The raw data supporting the conclusions of this article will be made available by the authors, without undue reservation.
